# The Emerging Influences of Alpha-Fetoprotein in the Tumorigenesis and Progression of Hepatocellular Carcinoma

**DOI:** 10.3390/cancers13205096

**Published:** 2021-10-12

**Authors:** Ye Xu, Qinglong Guo, Libin Wei

**Affiliations:** State Key Laboratory of Natural Medicines, Jiangsu Key Laboratory of Carcinogenesis and Intervention, School of Basic Medical Sciences and Clinical Pharmacy, China Pharmaceutical University, 24 Tongjiaxiang, Nanjing 210009, China; 3219091310@stu.cpu.edu.cn (Y.X.); 1019840800@cpu.edu.cn (Q.G.)

**Keywords:** hepatocellular carcinoma, alpha-fetoprotein, different functions in serum and cytoplasm

## Abstract

**Simple Summary:**

Hepatocellular carcinoma is characterized by aggressive invasion, rapid progress and poor prognosis. Since hepatocellular carcinoma is a highly heterogeneous cancer, both at the molecular and histological level, the precision medicine for patients is not very effective and the systematic targeted therapies remain unsatisfactory. The identification of new targets and molecular pathways is urgently needed for drug treatment of hepatocellular carcinoma. Alpha-fetoprotein acts as the most common tumor marker used for hepatocellular carcinoma diagnosis. Alpha-fetoprotein in serum and cytoplasm shows very different influences in the tumorigenesis and progression of hepatocellular carcinoma. The aim of this review is to summarize the current knowledge of the role of alpha-fetoprotein in hepatocellular carcinoma.

**Abstract:**

Hepatocellular carcinoma (HCC) is the sixth most common cancer worldwide, and its mortality rate is the third-highest, after lung cancer and colorectal cancer. Currently, systematic targeted therapies for HCC mainly include multiple kinase inhibitors and immunotherapy. However, these drugs carry a black-box warning about the potential for inducing severe toxicity, and they do not significantly prolong the survival period of patients due to the highly heterogeneous characteristics of HCC etiology. In order to improve the prediction, effective treatment and prognosis of HCC, the tools and different biomarkers in clinical practices are recommended. Alpha-fetoprotein (AFP) is the earliest and the most widely used serum marker in the detection of HCC. Interestingly, serum AFP and cytoplasmic AFP show different, even opposite, roles in the cancer progression of HCC. This review focuses on biological characteristics, regulatory mechanisms for gene expression, emerging influences of AFP in HCC and its possible implications in HCC-targeted therapy.

## 1. Introduction

The latest global cancer burden data of 2020 released by the International Cancer Research Institute of the World Health Organization (IARC) show that hepatocellular carcinoma (HCC) is the sixth most common cancer in the world and its mortality rate is the third-highest. In 2020, the number of new cases of liver cancer in the world was 91 million, and the number of deaths was 83 million [1]. Rates of both incidence and mortality are two to three times higher among men than among women in most regions. Recently, Fudan University published the research in the International Journal of Epidemiology, an international authoritative epidemiological journal. The model predicts that the incidence rate of global primary liver cancer will continue to rise as far as 2030 (reaching 14.08/10 million) [2]. The highest incidence rates are observed mainly in transitioning countries in Eastern Asia, South-Eastern Asia and Northern and Western Africa, while Northern Europe and South Central Asia have rates far lower than any other region [1,3]. China also shows the incidence rate of liver cancer and the burden of serious liver cancer. In 2020, 410 thousand new cases of primary liver cancer were found in China, and the number of deaths was the second among the top ten cancer deaths in China, reaching 390 thousand cases. The primary liver cancer cases in China account for 55% of cases worldwide, and the five-year survival rate was not significantly improved in the past two decades [4]. HCC contributes 75–85% of cases to all cases of primary liver cancer. The main risk factors for HCC are chronic infection with hepatitis B virus (HBV) or hepatitis C virus (HCV), aflatoxin-contaminated foodstuffs, heavy alcohol intake, obesity, smoking and type 2 diabetes [5]. In most cases, HCC develops in cirrhotic livers, and cirrhosis is the strongest risk factor for the disease. The composition of diet might be one clue to the elaboration of the mechanisms relating obesity, nonalcoholic fatty liver disease (NAFLD) and HCC [6]. IL-6 and TNFα expression in the liver is strongly induced in response to a high-fat diet, which can also increase nuclear factor-kappa B (NF-κB) activation [7]. De novo lipogenesis in obese, diabetic and NAFLD subjects amplifies lipotoxic injury [8]. Alcoholic liver cirrhosis is also considered to be an important risk factor for the development of HCC. It has been reported that approximately 10–20% of heavy drinkers develop steatosis, steatohepatitis and cirrhosis [9]. These pathological events are subsequently sustained and participate in the hepatic alcohol-mediated carcinogenetic process, such as the direct detrimental effects of metabolite acetaldehyde on proteins and DNA, the aggravated impairment of antioxidant defenses and DNA repair by reactive oxygen species (ROS), changes to the immune system and the induction of chronic inflammation [10].

Since HCC is characterized by untimely diagnosis, rapid progression, aggressive invasion and poor prognosis, the treatment for HCC has always been a difficult problem. Currently, potentially curative treatments for HCC include surgery (resection or transplant), radiofrequency ablation and percutaneous ethanol injection for patients with very early or early-stage HCC (only about 5–15%) [11,12,13,14] and transarterial chemoembolization for those with intermediate-stage HCC. However, patients with advanced HCC have limited treatment options. Sorafenib, a multi-targeted kinase inhibitor, is recommended worldwide as the first-line therapy for patients with advanced HCC [15]. Nevertheless, fewer than one-third of patients could benefit from Sorafenib, and drug resistance is severe within six months of initiating the regimen [16]. At present, natural compounds, chemotherapeutics, immunotherapies and new methods for delivery of drugs are increasing the possibility of treating HCC [17]. To further decrease disease mortality and increase patient survival, the tools and different biomarkers for better prediction and prognosis in clinical practices are recommended based on the risk levels [4,18]. Alpha-fetoprotein (AFP) has been used as a clinical biomarker to diagnose HCC since the 1970s [19]. More than 50% of HCC patients secrete AFP into the serum to provide an increased concentration of AFP of up to 0.5–1 mg/mL [20]. Hence, high serum levels of AFP usually represent the tumorigenesis and progression of HCC [21]. Although a report from the Affiliated Hospital of Qingdao University showed that serum AFP levels of patients suffering from breast, esophagus, cervical, pancreatic, gastric, lung and some other types of cancers were higher than those of healthy individuals (*p* < 0.05), AFP is not mature enough to be a biomarker for other types of cancers due to the unconventional and non-obvious correlation between the elevation of serum AFP levels and the incidence of other cancers. For example, the incidence of AFP-producing gastric cancer (GC) is approximately 1.3–15% in GC [22]. In the past two decades, there has been a great deal of research on AFP in both basic research and clinical practice. Before the 21st century, researchers put more emphasis on serum levels of AFP and their effect on the proliferation, migration, apoptosis and immune escape of normal and cancer cells. In the past decade, the research hotspot has gradually shifted to cellular AFP and its regulatory role in the occurrence and development of HCC. Interestingly, the two results of serum AFP and cytoplasmic AFP are not exactly the same. Therefore, figuring out the role of AFP in the malignant transformation and development of HCC is important for discovering new biological functions of AFP and molecular mechanisms involved in hepatocarcinogenesis, and AFP may become a potential bio-target for HCC treatment.

## 2. Biological Characteristics of AFP and Potential Biomarkers in Diagnosis of HCC

AFP is a mammalian tumor-associated fetal glycoprotein, with molecular weight 68–72 kDa which depends on the species of origin [23]. Its first discovery was by Bergstrand and Czar in human fetal serum in 1956 [24]. In terms of physiology, AFP is produced by the fetal liver and yolk sac during the first trimester of pregnancy, declines slightly during the second trimester, shows a rapid decline after birth and remains at low levels over the entire lifespan [24,25,26,27]. The normal serum levels of AFP during different stages mean healthy development of the fetus and normal physical function of the adult.

Serum levels of AFP that exceed those seen in healthy adults (5 ng/mL) have been reported in some patients with benign hepatic disorders, i.e., viral hepatitis or liver cirrhosis [28,29]. In 1963 and 1965, tumor-associated murine AFP and tumor-associated human AFP were detected separately by Abelev et al. and Tatarinov [30,31]. Considering the extremely aberrant AFP concentrations in the serum of patients with HCC, serum levels of AFP have been used as a clinical biomarker to diagnose HCC since the 1970s [19,21,32]. Although 70–90% of patients with HCC are AFP positive and its specificity could reach 72–90%, its sensitivity is only 9–32% [33]. Moreover, about 1/3 of the HCC patients show normal serum levels of AFP [34]. In order to diagnose HCC more accurately and earlier, more serum markers and tools have been added to the systemic model of HCC detection.

Over the last five years, in addition to physical methods, such as ultrasound (US)-based screening [18] and whole-body β-2-[18F]-Fluoro-2-deoxy-D-glucose positron emission tomography computer tomography (18F-FDG PET CT) [35], more and more biomarkers have been found to detect HCC, including heat shock protein 90α (HSP-90α) [36,37], *miR-132/212* [38], serum vitronectin (VN) [39], heterogeneous nuclear ribonucleoprotein H1 (hnRNPH1) mRNA [40], brain cytoplasmic RNA 1 (*BCYRN1*) [41], polymorphisms in the interleukin-18 promoter [42], methylation of SRY (sex-determining region Y)-box1 (*SOX1*) and vimentin promoters [43], lipopolysaccharide-binding protein (LBP) [44], insulin-like growth factor-binding protein 7 (*IGFBP7*) promoter methylation [45] and so on. These markers have been proven to improve the diagnostic efficiency for HCC combined with serum levels of AFP. Among these, des-γ-carboxy prothrombin (DCP) seems to have become a practical marker for the detection of HCC in view of its high specificity [46] and better prognosis for HCC. Many studies have compared the clinical usefulness of DCP and AFP as biomarkers for HCC, but the final conclusions are still being discussed. Considering its low sensitivity [47], DCP testing is currently approved only in Japan, South Korea and Indonesia [48]. However, multiple reports have shown that a combination of these two biomarkers is more effective in detecting HCC [49,50,51]. Japan has implemented a rigorous surveillance program relying on regular US surveillance with AFP and two additional biomarkers, lectin-reactive α-fetoprotein (AFP-L3) and DCP, which results in improvement of overall survival of HCC patients [52,53]. Meanwhile, there are some other biomarkers offsetting the defects of AFP. A novel bioassay for the Sonic Hedgehog (SHh) ligand in tissue specimens may help diagnose HCC with negative AFP and predict early microvascular invasion [54]. Serum annexin A3 (ANXA3) provides greater diagnostic performance than AFP, especially in early diagnosis and discriminating HCC from patients at risk [55] (Table 1). Despite the identification of candidate biomarkers, AFP is still the critical and central serum marker in the diagnosis of HCC [18,56].

## 3. Regulatory Mechanisms of AFP Expression

### 3.1. The Government of AFP Gene Transcription

The transcription of the *AFP* gene is regulated by five distinct regions, which are a 250-bp tissue-specific promoter, a 600-bp repressor region directly located upstream of the promoter [57] and three independent enhancers [58] also located upstream of the *AFP* promoter, named enhancer I, II and III. Since its transcription is regulated by five parts, the discordance of any part can be the cause of AFP overexpression. Here, AFP overexpression caused by the hereditary persistence of AFP (HAFP) is not considered, which will not result in any clinical abnormality [59,60].

For the promoter, low methylation is one reason for AFP overexpression. Robert and coworkers detected that the *AFP* promoter was in lower methylation in AFP-high tumors compared with the non-tumor-adjacent tissues and low-AFP-expression tumors [61]. In addition, the transcriptional regulators on the promoter are also attributed to the overexpression of AFP. Zinc finger and BTB domain containing 20 (ZBTB20) acts as a transcriptional repressor binding with mouse *AFP* promoter at −104/−86 bp [62]. Indeed, in model mice of liver-specific ZBTB20 knockout, ablation of ZBTB20 in postpartum liver leads to aberrant activation of *AFP* gene expression independent of hepatocyte proliferation [63], which might be related to the downregulation of epithelial growth factor receptor (EGFR) expression by deficiency of ZBTB20 [64]. Nevertheless, ZBTB20 expression is increased in patients with HCC. The abnormal elevation of ZBTB20 is associated with poor prognosis which is contrary to its role as a transcriptional repressor [65]. Kan and coworkers suggested that ZBTB20 may promote tumor growth of HCC through transcriptionally binding with the promoter of forkhead box O1 (*FoxO1*) to repress the expression of FoxO1 [66]. Zinc fingers and homeoboxes 2 (ZHX2) is another repressor acting on the *AFP* promoter to repress APF in both mice and humans [67]. However, a small study in Peruvian patients with HCC demonstrated that their expression of ZHX2 was enhanced [68]. In addition, T-complex protein 10A homolog 2 (TCP10L), a new-found repressor of *AFP* promoter, specifically expressed in the liver and testis in normal human tissues, is down-regulated in HCC [69].

As for the repression region, p53/ hepatocyte nuclear factor3 (HNF-3) binding site is critical in the regulation of the *AFP* gene. P53 acts as a developmental repressor of AFP in the liver by modifying the chromatin structure at the core promoter of the *AFP* gene rather than at local sites [70,71]. Nevertheless, p53 mutation resulting in aberrant AFP overexpression was detected in 41.5% of 325 HCC cases [72], suggesting p53 mutation is one of the reasons for AFP overexpression. In addition, hepatitis B virus X (HBx)-protein-driven AFP expression is also associated with p53. Ogden and coworkers showed that HBx induced expression of AFP by the physical interaction with p53, which blocked the interaction between p53 and other hepatic-specific proteins acting in transcription repression [73].

The regulation of the enhancer is also taken into consideration. Phosphoinositide-3-kinase regulatory subunit 3 (PIK3R3, also known as p55PIK), phosphatidylinositol 3 kinase (PI3K) regulatory isoform, was found to stimulate the transcription of the *AFP* gene by binding to the two NF-κB binding sites in the enhancer region (−5184/−1820), leading to the activation of the NF-κB signaling pathway [74].

### 3.2. Regulation of AFP/Receptor Autocrine and Paracrine Pathway

It has been found that AFP receptor (AFPR) is expressed in many kinds of malignant tumor cells, including HCC cells [75]. Meanwhile, the expression of AFPR and AFP are synchronous in HCC cells [76]. Li and coworkers [76] demonstrated positive feedback between AFP and AFPR that cytoplasmic AFP and AFPR were synchronously highly expressed in HBx-driven HCC cells through the activation of the PI3K/AKT (also known as protein kinase B, PKB) signal pathway. Combined with the conclusion that AFP promoted the proliferation of HCC cells [75], it is possible that this positive feedback of the AFP/AFPR autocrine pathway is involved in the overexpression of AFP.

In addition, paracrine stimulation of the *AFP* gene was found in some breast cancers. Peritumoral mammary adipocytes as well as fibroblasts and lymphocytes infiltrating the tumor, which expressed AFPR, were detected in the activation of the *AFP* gene and expression of the AFP mRNA transcripts [77]. Since HCC cells are surrounded with non-tumor stroma consisting of components of the extracellular matrix such as non-malignant fibroblasts and immune and endothelial cells [78], paracrine stimulation may exist in the development of HCC that enhances the expression of AFP. However, there is little research on this aspect, and more evidence is needed to prove it.

## 4. Effects of AFP on the Tumorigenesis and Progression of HCC

### 4.1. AFP and Cell Proliferation of HCC

In the past three decades, AFP has been found to have a biphasic effect of growth suppression and growth promotion in malignant and normal cells. High doses (more than 100 mg/L) of purified human AFP were shown to strongly inhibit the growth of human hepatoma HepG2 cells, human lymphoblastoma MT4 cells, lymphoma Jurkat cells and murine fibroblastoma L929 cells in a dose-dependent manner [79]. However, a low concentration of AFP (less than 100 mg/L) failed to induce growth inhibition of HepG2 cells, rather showing a weak stimulative effect [80]. Similarly, processing Bel 7402 cells with 20 mg/L AFP enhanced its proliferation, which might result from the induction of some oncogene expression, c-fos, N-ras, mutative p53 and p21 protein [81] and the stimulation of the cyclic adenosine monophosphate-protein kinase A(cAMP-PKA) pathway [75]. In most cases, extracellular AFP produces growth-regulatory effects by AFPR located in the cell membrane coupled with its transmembrane signaling transduction.

In addition to the concentration of AFP, growth factors and various cytokines could also influence the growth-regulating activity of AFP by some unknown mechanisms such as transmodulation (receptor cross-talk) [82]. For example, Interleukin-2 secretion was involved in the insensitivity of the Jurkat cell line to extracellular AFP-mediated cell death [83]. In contrast, exogenous AFP synergized epidermal growth factor (EGF) and insulin-like growth factor-I (IGF-I) to cause a marked increase in the proliferation of porcine granulosa cells [84]. It was also found that intracellular AFP was involved in the regulation of the PI3K/AKT signaling pathway during research on the drug resistance of HCC patients to all-trans retinoic acid (ATRA) via direct interaction with phosphatase and tensin homolog (PTEN) and competitive binding to the retinoic acid receptor β (RAR-β) which resulted in suppression of the *PTEN* gene and the enhancement of cell growth [85,86].

### 4.2. AFP and Cancer Cell Apoptosis

In the last decade, the research on AFP has gradually shifted from the circulating level to the cytoplasmic level. Interestingly, the effect of circulating and intracellular AFP on apoptosis in HCC cells is not exactly the same.

In the beginning, researchers paid more attention to circulating AFP and studied its function by exogenous addition to the culture medium. Semenkova and coworkers proposed that a high dose of exogenous addition of AFP derived from different sources (cord serum and culture medium of AFP-secreting HepG2 cells) can induce dose-dependent apoptosis of human hepatoma HepG2 cells [80]. The classical features of apoptosis were dose-dependent, appearing for various types of tumor cells, such as Raji, Jurkat and MCF-7 cells, at AFP concentrations of 0.7–3.0 μM and, more significantly, at 5.0–10.0 μM [87]. Further research found that this type of circulating AFP-induced apoptosis was independent of the Fas/Fas ligand (Fas/Fas-L) or tumor necrosis factor receptor/tumor necrosis factor (TNFR/TNF) signaling pathways and did not activate the upstream initiator caspases through activation of the effector caspase-3-like proteases [87]. On the other hand, it has been reported that extracellular and membrane-bound AFP can prevent TNF-induced cytotoxicity and apoptosis of HepG2 cells [88].

Over the last two decades, a cell-free system was often used to simulate the cytoplasmic environment in order to simplify the experimental process. Semenkova [89] and Dudich [90], respectively, demonstrated that AFP interacted with the inhibitor of apoptosis protein 2 (IAP-2) and X-linked inhibitor of apoptosis protein (XIAP) molecule, abolished IAP caspase binding and rescued caspase-3 from inhibition, leading to triggering of cytochrome c-mediated apoptosis.

Recently, more and more evidence has shown that intracellular AFP could inhibit the apoptosis of tumor cells. Moreover, the results come from the real intracellular environment which is more credible. In Huh7 cells, silencing AFP reduced the expression of mutant p53, leading to the change of the Bax/Bcl-2 ratio which triggered the release of cytochrome c from mitochondria into the cytosol and eventually induced apoptosis [91]. At present, studies have found that intracellular AFP-mediated apoptosis inhibition is mainly related to the interaction with several proteins, including caspase-3. In Bel 7402 cells, caspase-3 co-localized and interacted with AFP in the cytoplasm, which interfered with the translocation of caspase-3 into nuclei [92]. The structural basis for AFP-mediated inhibition of caspase-3 activity by interacting with caspase-3 loop-4 amino acid residues Glu-248, Asp-253 and His-257 was detected by Lin and coworkers [93]. Since its interaction with caspase-3, AFP eliminated apoptotic activity of the TNF-related apoptosis-inducing ligand (TRAIL) or TNF-induced in Bel 7402 cells [94] and HepG2 cells [80], respectively.

Despite that a variety of studies have shown different effects of AFP on cell growth regulation, the exact mechanisms between proliferation, apoptosis and AFP remain little known and controversial.

### 4.3. Immuno-Suppressive Activity of AFP

Previously, it was assumed that the fetus protects itself from the maternal immune system attack by secreting AFP into maternal circulation [95,96]. Furthermore, the immuno-suppressive activity of AFP may contribute to the occurrence of some malignant conditions. It is confirmed that immune escape of HCC cells is merely a consequence of the effects on the dendritic cells (DCs), natural killer cells (NK) and thymus-dependent lymphocytes (T lymphocytes). It was reported that treatment of monocyte-derived DCs with AFP (as low as 2.5 mg/L) induces DC dysfunction as detected by the downregulation of surface molecules and inhibition of their T-cell-stimulatory capacity. Meanwhile, AFP treatment reduced the ability of monocyte-derived DCs to produce TNF-α and IL-12 and induced apoptosis of DCs [97]. AFP has also been reported to inhibit the proliferative ability of NK cells and T lymphocytes. AFP-induced overexpression of Fas on the surface of lymphocytes, together with simultaneous over-secreted Fas-L from tumor cells, such as Bel 7402 cells, could be one of the reasons to accelerate the death of lymphocytes and facilitate the immune escape of liver cancers [98]. In addition, in SMCC 7721 cells, upregulation of cellular AFP increased the expression of PD-L1 and B7-H4 both in the level of mRNA and protein, which might promote tumor immune escape, possibly via activation of the NF-κB pathway [99]. In addition, one report from South Egypt Cancer Institute showed that serum levels of AFP are positively correlated with the percentage of regulatory T cells in peripheral blood [100].

In brief, the immuno-suppressive effect of AFP is through two indispensable ways: on the one hand, extracellular AFP induces the apoptosis of immune cells and attenuates their antitumor function, but its specific mechanism needs to be further explored. It has been reported that AFPR also exists in the transmembrane of immune cells, such as T lymphocytes [101], and there is a paracrine AFP/AFPR system, which may mediate the effect between AFP and immune cells. On the other hand, extracellular AFP promotes tumor cells to secrete ligands such as Fas-L, while intracellular AFP promotes the expression of surface antigens such as PD-L1 and B7-H4, so as to achieve the role of immune escape.

### 4.4. AFP and Invasion, Metastasis and Angiogenesis of HCC

Extrahepatic metastasis occurs in one-third of patients with HCC [102,103]. The most common sites for HCC’s extrahepatic metastasis are lung, lymph nodes, bone and adrenal glands [104,105]. Serum AFP, tumor size and vascular invasion are strongly associated with extrahepatic metastasis of HCC [106]. Considering the high incidence of metastasis after liver transplantation, serum AFP levels have usually been used as a monitoring indicator for metastasis before and after the transplant surgery [107,108]. To improve the sensitivity and specificity for predicting the recurrence and metastasis of HCC, detection of AFP mRNA in circulating tumor cells (CTCs) and traveling cells in physiological fluids released from a primary or metastatic tumor can be an alternative method for the imaging and detection of serum AFP levels in that the imaging is too expensive and has size limitations for the metastasis tumor and serum AFP monitoring has delayed effect [109]. In vitro, transient changes of cytoplasmic AFP expression in Bel 7402 or HLE cells could affect the expression of metastasis-related proteins, keratin 19(K19), epithelial cell adhesion molecule(EpCAM), matrix metalloproteinase 2/9 (MMP2/9) and C-X-C motif chemokine receptor 4 (CXCR4) [110], which are also markers of stem cells. The results demonstrate that AFP plays a critical role in promoting the metastasis of HCC. Immune escape via increase of the expression of PD-L1 and B7-H4 could also be one way of AFP strengthening the ability of metastasis of HCC cells [99]. In vivo, after orthotopic implantation of tumor tissue in nude mice, serum AFP levels were 246 ± 66 μg/L for MHCC97-H with high metastatic potential and 91 ± 66 μg/L for MHCC97-L with low metastatic potential (*p* < 0.01, *t* test) [111]. Thus, most studies have proven that AFP is involved in the promotion of metastasis and invasion of HCC.

In addition to the pro-metastatic ability of tumor cells, angiogenesis around tumor tissue is another important guarantee for tumor cell invasion and metastasis. Research assessing transcriptome data, whole-exome sequencing data and DNA methylome profiling of 520 HCC patients demonstrated that AFP-high (serum concentration > 400 mg/L) tumors displayed significant activation of vascular endothelial growth factor (VEGF) signaling [61]. In gastric cancer, VEGF expression may contribute to the higher microvessel density of the AFP-producing gastric cancers than that of the AFP-negative ones [112]. These findings demonstrate that AFP expression is associated with potentially more angiogenic tumors.

## 5. Summary

Serum AFP levels are the earliest biomarker used in the detection of HCC. However, it is not the most optimal criterion for the detection and prognosis of HCC. Thus, much more biomarkers and physical tools are added to the criterion to improve the specificity for HCC and universality for AFP-negative HCC.

**Figure 1:**
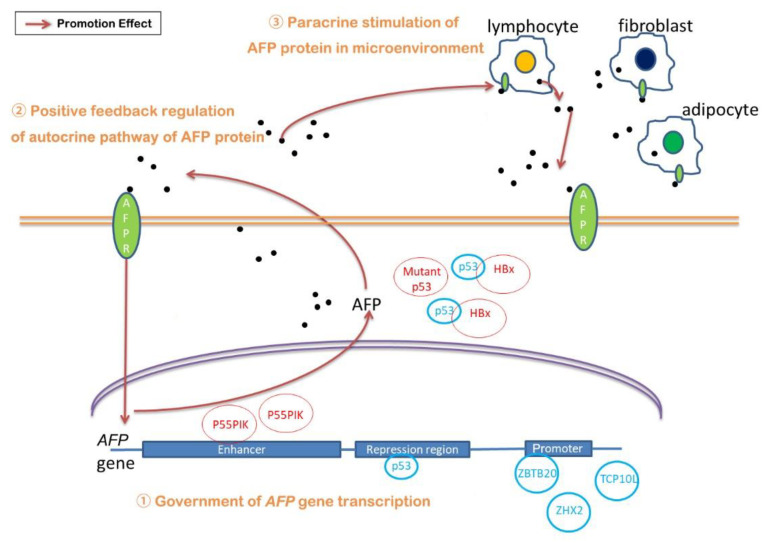
Regulatory mechanisms of AFP expression. (**1**) Government of *AFP* gene transcription, (**2**) positive feedback regulation of autocrine pathway of AFP protein and (**3**) paracrine stimulation of AFP in microenvironment.

The overexpression of AFP seems to be a very complex process, and many studies have been carried out on its mechanism (Figure 1). (1) The regulation of different transcription factors on different locations of the *AFP* gene, including promoter, repression region and enhancer, affects the expression of AFP. Among these transcription factors, it is worth noting the role of p53, which seems to be closely related to the occurrence of many cancers. (2) The positive feedback of the AFP/AFPR autocrine pathway increased the expression of AFP. (3) Meanwhile, the paracrine pathway formed by tumor cells and its surrounding cells, such as mammary adipocytes, also increased AFP expression to some extent. However, there are not many reports on this pathway, except for breast cancers. Considering that adipocytes, infiltrating lymphocytes and fibroblasts in the tumor microenvironment have a great impact on HCC, it is necessary to further verify whether this paracrine pathway exists in HCC. These three aspects play a role at the same time, resulting in the overexpression of AFP. Meanwhile, what causes the regulation of those transcription factors influencing the regulation of the *AFP* gene and the proportion of these three aspects all need to be further studied.

**Figure 2:**
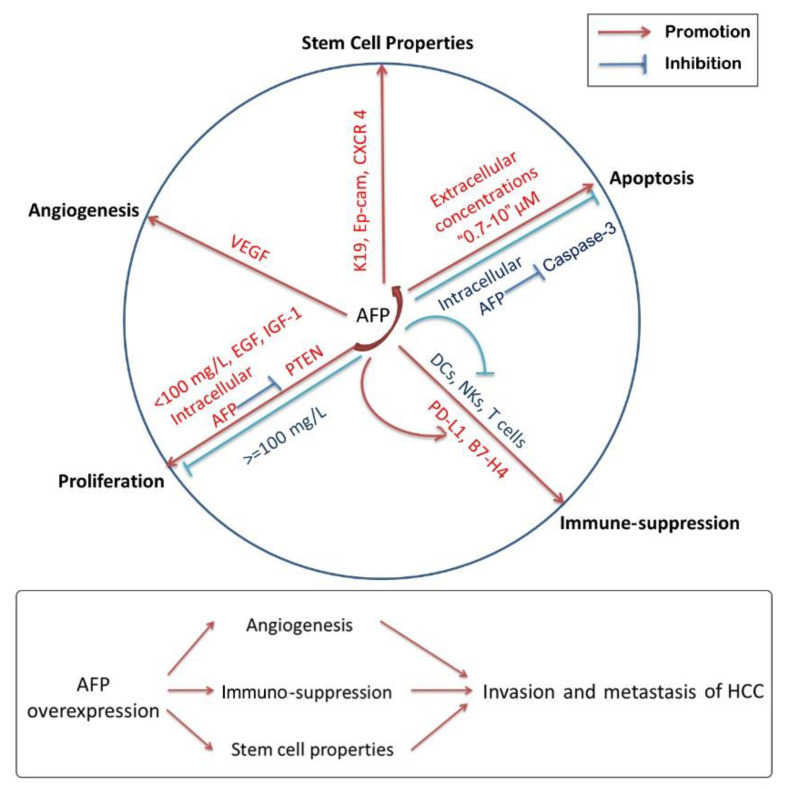
Effects of AFP on the tumorigenesis and progression of HCC. AFP shows multi-effects on HCC, including cell proliferation, cell apoptosis, tumor immunology, stem cell properties, angiogenesis and so on. Overexpression of AFP promotes the invasion and metastasis of HCC via the immuno-suppression and the improvement of stem cell properties as well as angiogenesis. Interestingly, AFP has complicated controversial effects in cell proliferation and apoptosis according to the concentration of AFP and the status of AFP that is circulating or cytoplasmic.

It took nearly ten years to change researchers’ focus from circulating AFP to cytoplasmic AFP. At present, most studies have found that cytoplasmic AFP can promote the occurrence of HCC by promoting the proliferation of cancer cells, inhibiting apoptosis and inducing immuno-suppression. In addition, it also plays a significant role in enhancing the invasion, metastasis and angiogenesis of HCC cells (Figure 2). However, as shown in Table 2, circulating AFP and cytoplasmic AFP have different effects on proliferation, apoptosis, immuno-suppression, stem cell properties and angiogenesis. Even in terms of apoptosis, the same role of circulating or cytoplasmic AFP is opposite. Since the results are complex and controversial, determining the causal relationship between AFP abnormal expression and other carcinogenic factors is the key to revealing the true nature of AFP.

## 6. Prospect of AFP-Targeted Therapies

Considering the uncertainty of AFP function in the tumorigenesis and progression of HCC cells, whether it can be used as a therapeutic target for liver cancer is controversial. Combined with the current research, the use of antibodies to AFP in cancer is not very practical. Even when circulating serum AFP levels were effectively suppressed, complete tumor ablation was not achieved, and subsequent attempts to confirm the effectiveness of antiAFP antibodies were only a partial success [113]. There are many reasons for the failure, such as the use of polyclonal antibodies, the rapid clearance of heterologous antibodies and so on. Among these, the complexity of the AFP function greatly contributes to the failure. Compared with these kinds of antibodies to AFP, the use of AFP as an anticancer drug conjugate by taking advantage of the specific expression of AFP in tumor cells is more feasible. A variety of anticancer drugs showed enhanced therapeutic activity when conjugated with AFP, such as carminomycin, 2,3,7,8-tetrachlorodibenzo-p-dioxin and doxorubicin [114,115,116]. In addition, for gene therapy, utilization of tumor-specific activity of an *AFP* gene promoter sequence could induce hepatoma cells to be more sensitive to ganciclovir by initiating the expression of herpes simplex virus thymidine kinase gene, a kind of exogenous gene under the control of the 0.3 kb human *AFP* gene promoter [117]. Nowadays, genetic modification with T-cell receptors (TCRs) specific for AFP can potentially redirect TCR-gene-engineered human T cells to specifically recognize and then kill HCC tumor cells [118,119,120]. Certainly, a large number of studies are needed to provide more reliable evidence.

## 7. Conclusions

Taken together, the above review indicates a comprehensive knowledge of the molecular regulations and functions of AFP during HCC tumorigenesis and progression. This information not only has an impact on the overall survival of patients but also provides potential insight into targeted therapies.

## Figures and Tables

**Table 1:** Potential diagnostic biomarkers and their influences on HCC.

Potential Diagnostic Biomarker	Molecular Functions	Diagnostic Application for HCC	Reference
HSP-90α	A molecular chaperone; is associated with the folding and unfolding of diverse proteins, such as signaling protein kinases, transcription factors.	Plasma HSP-90α levels in HCC group were significantly higher than that in the healthy control group and the benign liver disorder group, which did not depend on the origin of the patients with HCC. Combined with AFP and thymidine kinase 1(TK1), the detection of HSP90-α increased the sensitivity for the diagnosis of HCC.	[36,37]
*miR-132/212*	Dysregulated in many human malignancies and plays important roles in tumor progression.	*miR-132* and *miR-212* are downregulated in HCC and cell lines, as tumor-suppressive roles. Combination of serum *miR-132/212* cluster and AFP markedly improved sensitivity of diagnosis of HCC, compared with AFP or miR-132/212 alone.	[38]
Serum VN	A cell-adhesive glycoprotein in serum and plasma; is associated with inflammation, cell adhesion, cell necrosis, angiogenesis, etc.	Serum VN levels were significantly elevated in HBV-related HCC patients. Serum VN levels had similar diagnostic and prognostic values compared with serum AFP levels in HBV-positive HCC patients.	[39]
hnRNPH1 mRNA	As a kind of RNA binding protein, hnRNPH1 is associated with pre-mRNAs and appears to influence pre-mRNA processing and other aspects of mRNA metabolism and transport. It is highly expressed in many kinds of cancers.	The mRNA levels of hnRNPH1 in serum of HCC patients were remarkably higher than those in normal individuals, which were positively associated with Child–Pugh classification, portal vein tumor emboli, lymph node metastasis and tumor node metastasis (TNM) stage. It may be a novel biomarker for diagnosis of HCC in high-HBV-prevalence areas.	[40]
*BCYRN1*	A type of long noncoding RNA; acts as a competing endogenous RNA, regulating cell migration, proliferation and survival.	*BCYRN1* expression was elevated in HCC patients. It suggested that the diagnosis of HCC was remarkably improved with the combination of plasma *BCYRN1* and AFP.	[41]
LBP	A serum protein, synthesized in the liver; is involved in the recognition, binding and transport of the bacterial cell wall compound lipopolysaccharide/endotoxin.	LBP is overexpressed in HCC tissues, especially in poorly differentiated tumors.	[44]
DCP	An abnormal prothrombin precursor produced in HCC; lacks the ability to interact with other coagulation factors.	In 63% of patients with HCC, serum DCP levels were found to be elevated. Combination of AFP and 2 additional biomarkers, AFP-L3 and DCP, results in improvement of overall survival of HCC patients.	[52,53]
SHh ligand	A unique signaling molecule in hedgehog signaling; controls embryonic development and tissue repair under physiological conditions.	The upregulation of SHh ligand was frequently observed in HCC and was greatly associated with drug resistance and metastasis of the malignancy through epithelial-to-mesenchymal transition (EMT). It could also be compensatory for detection of AFP-negative HCC patients.	[54]
Serum ANXA3	A member of calcium-dependent phospholipid-binding protein family; participates in a diverse range of physiological activities that include anticoagulation, anti-inflammatory activities, endocytosis and exocytosis, signal transduction, cell proliferation, cell differentiation and cell apoptosis.	ANXA3 expression is significantly unregulated in patients with HCC. Clinically, ANXA3 expression in HCC patients sera and tissues is closely associated with aggressive clinical features. Serum ANXA3 provides greater diagnostic performance than AFP, especially in early diagnosis and discriminating HCC from patients at risk.	[55]

**Table 2:** Comparison between circulating (extracellular) and cytoplasmic (intracellular) AFP on the effects of the tumorigenesis and progression of HCC.

Regulations	Effects	Circulating (Extracellular) AFP	Cytoplasmic (Intracellular) AFP
Proliferation	Promotion	High doses (more than 100 mg/L) of purified human AFP induced dose-dependent growth inhibition.	Intracellular AFP was involved in the regulation of PI3K/AKT via the direct interaction with PTEN which resulted in suppression of the *PTEN* gene and enhancement of cell growth.
	Inhibition	Low concentration of AFP (less than 100 mg/L) showed a weak stimulative growth effect in HepG2 cells.	None.
Apoptosis	Induction	The induction of apoptosis was dose-dependent in various types of tumor cells, such as Raji, Jurkat and MCF-7 cells, at AFP concentrations of 0.7–3.0 μM and, more significantly, at 5.0–10.0 μM.	AFP interacted with IAP-2 and XIAP, abolished IAP caspase binding and rescued caspase-3 from inhibition, triggering cytochrome c-mediated apoptosis.
	Inhibition	Extracellular and membrane-bound AFP prevented TNF-induced cytotoxicity and apoptosis of HepG2 cells.	Since its interaction with caspase-3, AFP eliminated apoptotic activity of TRAIL or TNF-induced in Bel 7402 cells and HepG2 cells, respectively.
Immuno-suppression	For tumor cells	Extracellular AFP promotes tumor cells to secrete ligands such as Fas-L.	Intracellular AFP promotes the expression of surface antigens such as PD-L1 and B7-H4, so as to achieve the role of immune escape.
	For immune cells	Extracellular AFP induces the apoptosis of immune cells and attenuates their antitumor function.	None.
Stem cell properties	Promotion	None.	Transient changes of AFP expression in Bel 7402 or HLE cells could regulate the expression of K19, EpCAM and CXCR4, which are markers of stem cells.
Angiogenesis	Positive relation	Whole-exome sequencing data and DNA methylome profiling of 520 HCC patients demonstrated that AFP-high (serum concentration > 400 mg/L) tumors displayed significant activation of VEGF signaling.	None.

## References

[1] Sung H., Ferlay J., Siegel R.L., Laversanne M., Soerjomataram I., Jemal A., Bray F. (2021). Global Cancer Statistics 2020: GLOBOCAN Estimates of Incidence and Mortality Worldwide for 36 Cancers in 185 Countries. CA Cancer J. Clin..

[2] Liu Z., Xu K., Jiang Y., Cai N., Fan J., Mao X., Suo C., Jin L., Zhang T., Chen X. (2021). Global trend of aetiology-based primary liver cancer incidence from 1990 to 2030: A modelling study. Int. J. Epidemiol..

[3] Choo S.P., Tan W.L., Goh B., Tai W.M., Zhu A.X. (2016). Comparison of hepatocellular carcinoma in Eastern versus Western populations. Cancer.

[4] (2021). Guideline for stratified screening and surveillance of primary liver cancer(2020 Edition). Zhonghua Zhong Liu Za Zhi.

[5] Bray F., Ferlay J., Soerjomataram I., Siegel R.L., Torre L.A., Jemal A. (2018). Global cancer statistics 2018: GLOBOCAN estimates of incidence and mortality worldwide for 36 cancers in 185 countries. CA Cancer J. Clin..

[6] Marengo A., Rosso C., Bugianesi E. (2016). Liver Cancer: Connections with Obesity, Fatty Liver, and Cirrhosis. Annu. Rev. Med..

[7] Vanni E., Bugianesi E. (2014). Obesity and liver cancer. Clin. Liver Dis..

[8] Donnelly K.L., Smith C.I., Schwarzenberg S.J., Jessurun J., Boldt M.D., Parks E.J. (2005). Sources of fatty acids stored in liver and secreted via lipoproteins in patients with nonalcoholic fatty liver disease. J. Clin. Investig..

[9] Matsushita H., Takaki A. (2019). Alcohol and hepatocellular carcinoma. BMJ Open Gastroenterol..

[10] Taniai M. (2020). Alcohol and hepatocarcinogenesis. Clin. Mol. Hepatol..

[11] Tsochatzis E., Meyer T., O’Beirne J., Burroughs A.K. (2013). Transarterial chemoembolisation is not superior to embolisation alone: The recent European Association for the Study of the Liver (EASL) European Organisation for Research and Treatment of Cancer (EORTC) guidelines. Eur. J. Cancer.

[12] Bruix J., Sherman M., American Association for the Study of Liver Diseases (2011). Management of hepatocellular carcinoma: An update. Hepatology.

[13] Shiina S., Tateishi R., Arano T., Uchino K., Enooku K., Nakagawa H., Asaoka Y., Sato T., Masuzaki R., Kondo Y. (2012). Radiofrequency ablation for hepatocellular carcinoma: 10-year outcome and prognostic factors. Am. J. Gastroenterol..

[14] Shiina S., Tateishi R., Imamura M., Teratani T., Koike Y., Sato S., Obi S., Kanai F., Kato N., Yoshida H. (2012). Percutaneous ethanol injection for hepatocellular carcinoma: 20-year outcome and prognostic factors. Liver Int..

[15] Vogel A., Cervantes A., Chau I., Daniele B., Llovet J.M., Meyer T., Nault J.C., Neumann U., Ricke J., Sangro B. (2018). Hepatocellular carcinoma: ESMO Clinical Practice Guidelines for diagnosis, treatment and follow-up. Ann. Oncol..

[16] El-Serag H.B., Marrero J.A., Rudolph L., Reddy K.R. (2008). Diagnosis and treatment of hepatocellular carcinoma. Gastroenterology.

[17] Anwanwan D., Singh S.K., Singh S., Saikam V., Singh R. (2020). Challenges in liver cancer and possible treatment approaches. Biochim. Biophys. Acta Rev. Cancer.

[18] Heimbach J.K., Kulik L.M., Finn R.S., Sirlin C.B., Abecassis M.M., Roberts L.R., Zhu A.X., Murad M.H., Marrero J.A. (2018). AASLD guidelines for the treatment of hepatocellular carcinoma. Hepatology.

[19] Kumada T., Nakano S., Takeda I., Kiriyama S., Sone Y., Hayashi K., Katoh H., Endoh T., Sassa T., Satomura S. (1999). Clinical utility of Lens culinaris agglutinin-reactive alpha-fetoprotein in small hepatocellular carcinoma: Special reference to imaging diagnosis. J. Hepatol..

[20] Butterfield L.H., Economou J.S., Gamblin T.C., Geller D.A. (2014). Alpha fetoprotein DNA prime and adenovirus boost immunization of two hepatocellular cancer patients. J. Transl. Med..

[21] Bai D.S., Zhang C., Chen P., Jin S.J., Jiang G.Q. (2017). The prognostic correlation of AFP level at diagnosis with pathological grade, progression, and survival of patients with hepatocellular carcinoma. Sci. Rep..

[22] Sun W., Liu Y., Shou D., Sun Q., Shi J., Chen L., Liang T., Gong W. (2015). AFP (alpha fetoprotein): Who are you in gastrology?. Cancer Lett..

[23] Mizejewski G.J. (1995). The phylogeny of alpha-fetoprotein in vertebrates: Survey of biochemical and physiological data. Crit. Rev. Eukaryot Gene Expr..

[24] Bergstrand C.G., Czar B. (1956). Demonstration of a new protein fraction in serum from the human fetus. Scand. J. Clin. Lab. Investig..

[25] Obiekwe B.C., Malek N., Kitau M.J., Chard T. (1985). Maternal and fetal alphafetoprotein (AFP) levels at term. Relation to sex, weight and gestation of the infant. Acta Obstet. Gynecol. Scand..

[26] Waller D.K., Lustig L.S., Cunningham G.C., Feuchtbaum L.B., Hook E.B. (1996). The association between maternal serum alpha-fetoprotein and preterm birth, small for gestational age infants, preeclampsia, and placental complications. Obstet. Gynecol..

[27] Bader D., Riskin A., Vafsi O., Tamir A., Peskin B., Israel N., Merksamer R., Dar H., David M. (2004). Alpha-fetoprotein in the early neonatal period-a large study and review of the literature. Clin. Chim. Acta.

[28] Ballas M. (1972). Yolk sac carcinoma of the ovary with alpha fetoprotein in serum and ascitic fluid demonstrated by immunoosmophoresis. Am. J. Clin. Pathol..

[29] Miyazaki J., Endo Y., Oda T. (1981). Lectin affinities of alpha-fetoprotein in liver cirrhosis, hepatocellular carcinoma and metastatic liver tumor. Kanzo.

[30] Abelev G.I., Perova S.D., Khramkova N.I., Postnikova Z.A., Irlin I.S. (1963). Production of embryonal alpha-globulin by transplantable mouse hepatomas. Transplantation.

[31] Tatarinov Y.S. (1966). Content of embryo-specific alpha-globulin in fetal and neonatal sera and sera from adult humans with primary carcinoma of the liver. Fed. Proceedings. Transl. Suppl. Sel. Transl. Med.-Relat. Sci..

[32] Bellissimo F., Pinzone M.R., Cacopardo B., Nunnari G. (2015). Diagnostic and therapeutic management of hepatocellular carcinoma. World J. Gastroenterol..

[33] Tsuchiya N., Sawada Y., Endo I., Saito K., Uemura Y., Nakatsura T. (2015). Biomarkers for the early diagnosis of hepatocellular carcinoma. World J. Gastroenterol..

[34] Zong J., Fan Z., Zhang Y. (2020). Serum Tumor Markers for Early Diagnosis of Primary Hepatocellular Carcinoma. J. Hepatocell. Carcinoma.

[35] Ali S.A., Amin D.H., Abdelkhalek Y.I. (2020). Efficiency of whole-body 18F-FDG PET CT in detecting the cause of rising serum AFP level in post-therapeutic follow-up for HCC patients. Jpn. J. Radiol..

[36] Qin L., Huang H., Huang J., Wang G., Huang J., Wu X., Li J., Yi W., Liu L., Huang D. (2019). Biological characteristics of heat shock protein 90 in human liver cancer cells. Am. J. Transl. Res..

[37] Tang Y., Li K., Cai Z., Xie Y., Tan X., Su C., Li J. (2020). HSP90α combined with AFP and TK1 improved the diagnostic value for hepatocellular carcinoma. Biomark. Med..

[38] Wang F., Wang J., Ju L., Chen L., Cai W., Yang J. (2018). Diagnostic and prognostic potential of serum miR-132/212 cluster in patients with hepatocellular carcinoma. Ann. Clin. Biochem..

[39] Yang X.P., Zhou L.X., Yang Q.J., Liu L., Cai Y., Ma S.L. (2016). Diagnostic and prognostic roles of serum vitronectin in hepatitis B-related hepatocellular carcinoma. Cancer Biomark..

[40] Xu H., Dong X., Chen Y., Wang X. (2018). Serum exosomal hnRNPH1 mRNA as a novel marker for hepatocellular carcinoma. Clin. Chem. Lab. Med..

[41] Ming X.L., Feng Y.L., He D.D., Luo C.L., Rong J.L., Zhang W.W., Ye P., Chai H.Y., Liang C.Z., Tu J.C. (2019). Role of BCYRN1 in hepatocellular carcinoma pathogenesis by lncRNA-miRNA-mRNA network analysis and its diagnostic and prognostic value. Epigenomics.

[42] Bakr N.M., Awad A., Moustafa E.A. (2018). Association of genetic variants in the interleukin-18 gene promoter with risk of hepatocellular carcinoma and metastasis in patients with hepatitis C virus infection. IUBMB Life.

[43] Liu X.Y., Fan Y.C., Gao S., Zhao J., Chen L.Y., Li F., Wang K. (2017). Methylation of SOX1 and VIM promoters in serum as potential biomarkers for hepatocellular carcinoma. Neoplasma.

[44] Cai Q.Y., Jiang J.H., Jin R.M., Jin G.Z., Jia N.Y. (2020). The clinical significance of lipopolysaccharide binding protein in hepatocellular carcinoma. Oncol. Lett..

[45] Tao L.P., Fan X.P., Fan Y.C., Zhao J., Gao S., Wang K. (2018). Combined detection of insulin-like growth factor-binding protein 7 promoter methylation improves the diagnostic efficacy of AFP in hepatitis B virus-associated hepatocellular carcinoma. Pathol. Res. Pract..

[46] Fujiyama S., Morishita T., Hashiguchi O., Sato T. (1988). Plasma abnormal prothrombin (des-gamma-carboxy prothrombin) as a marker of hepatocellular carcinoma. Cancer.

[47] Ikoma J., Kaito M., Ishihara T., Nakagawa N., Kamei A., Fujita N., Iwasa M., Tamaki S., Watanabe S., Adachi Y. (2002). Early diagnosis of hepatocellular carcinoma using a sensitive assay for serum des-gamma-carboxy prothrombin: A prospective study. Hepatogastroenterology.

[48] Inagaki Y., Tang W., Makuuchi M., Hasegawa K., Sugawara Y., Kokudo N. (2011). Clinical and molecular insights into the hepatocellular carcinoma tumour marker des-γ-carboxyprothrombin. Liver. Int..

[49] Ishii M., Gama H., Chida N., Ueno Y., Shinzawa H., Takagi T., Toyota T., Takahashi T., Kasukawa R. (2000). Simultaneous measurements of serum alpha-fetoprotein and protein induced by vitamin K absence for detecting hepatocellular carcinoma. South. Tohoku District Study Group. Am. J. Gastroenterol..

[50] Shimauchi Y., Tanaka M., Kuromatsu R., Ogata R., Tateishi Y., Itano S., Ono N., Yutani S., Nagamatsu H., Matsugaki S. (2000). A simultaneous monitoring of Lens culinaris agglutinin A-reactive alpha-fetoprotein and des-gamma-carboxy prothrombin as an early diagnosis of hepatocellular carcinoma in the follow-up of cirrhotic patients. Oncol. Rep..

[51] Durazo F.A., Blatt L.M., Corey W.G., Lin J.H., Han S., Saab S., Busuttil R.W., Tong M.J. (2008). Des.-gamma-carboxyprothrombin, alpha-fetoprotein and AFP-L3 in patients with chronic hepatitis, cirrhosis and hepatocellular carcinoma. J. Gastroenterol. Hepatol..

[52] Johnson P., Berhane S., Kagebayashi C., Satomura S., Teng M., Fox R., Yeo W., Mo F., Lai P., Chan S.L. (2017). Impact of disease stage and aetiology on survival in hepatocellular carcinoma: Implications for surveillance. Br. J. Cancer.

[53] Singal A.G., El-Serag H.B. (2015). Hepatocellular Carcinoma From Epidemiology to Prevention: Translating Knowledge into Practice. Clin. Gastroenterol. Hepatol..

[54] Ding J., Li H.Y., Zhang L., Zhou Y., Wu J. (2021). Hedgehog Signaling, a Critical Pathway Governing the Development and Progression of Hepatocellular Carcinoma. Cells.

[55] Ma X.L., Jiang M., Zhao Y., Wang B.L., Shen M.N., Zhou Y., Zhang C.Y., Sun Y.F., Chen J.W., Hu B. (2018). Application of Serum Annexin A3 in Diagnosis, Outcome Prediction and Therapeutic Response Evaluation for Patients with Hepatocellular Carcinoma. Ann. Surg. Oncol..

[56] Hughes D.M., Berhane S., de Groot C.A.E., Toyoda H., Tada T., Kumada T., Satomura S., Nishida N., Kudo M., Kimura T. (2021). Serum Levels of α-Fetoprotein Increased More Than 10 Years Before Detection of Hepatocellular Carcinoma. Clin. Gastroenterol. Hepatol..

[57] Vacher J., Tilghman S.M. (1990). Dominant negative regulation of the mouse alpha-fetoprotein gene in adult liver. Science.

[58] Lazarevich N.L. (2000). Molecular mechanisms of alpha-fetoprotein gene expression. Biochemistry.

[59] Jeon Y., Choi Y.S., Jang E.S., Kim J.W., Jeong S.H. (2017). Persistent α-Fetoprotein Elevation in Healthy Adults and Mutational Analysis of α-Fetoprotein Promoter, Enhancer, and Silencer Regions. Gut. Liver..

[60] Nagata-Tsubouchi Y., Ido A., Uto H., Numata M., Moriuchi A., Kim I., Hasuike S., Nagata K., Sekiya T., Hayashi K. (2005). Molecular mechanisms of hereditary persistence of alpha-fetoprotein (AFP) in two Japanese families A hepatocyte nuclear factor-1 site mutation leads to induction of the *AFP* gene expression in adult livers. Hepatol. Res..

[61] Montal R., Andreu-Oller C., Bassaganyas L., Esteban-Fabró R., Moran S., Montironi C., Moeini A., Pinyol R., Peix J., Cabellos L. (2019). Molecular portrait of high alpha-fetoprotein in hepatocellular carcinoma: Implications for biomarker-driven clinical trials. Br. J. Cancer.

[62] Zhang H., Cao D., Zhou L., Zhang Y., Guo X., Li H., Chen Y., Spear B.T., Wu J.W., Xie Z. (2015). ZBTB20 is a sequence-specific transcriptional repressor of alpha-fetoprotein gene. Sci. Rep..

[63] Xie Z., Zhang H., Tsai W., Zhang Y., Du Y., Zhong J., Szpirer C., Zhu M., Cao X., Barton M.C. (2008). Zinc finger protein ZBTB20 is a key repressor of alpha-fetoprotein gene transcription in liver. Proc. Natl. Acad. Sci. USA.

[64] Zhang H., Shi J.H., Jiang H., Wang K., Lu J.Y., Jiang X., Ma X., Chen Y.X., Ren A.J., Zheng J. (2018). ZBTB20 regulates EGFR expression and hepatocyte proliferation in mouse liver regeneration. Cell Death Dis..

[65] Wang Q., Tan Y.X., Ren Y.B., Dong L.W., Xie Z.F., Tang L., Cao D., Zhang W.P., Hu H.P., Wang H.Y. (2011). Zinc finger protein ZBTB20 expression is increased in hepatocellular carcinoma and associated with poor prognosis. BMC Cancer.

[66] Kan H., Huang Y., Li X., Liu D., Chen J., Shu M. (2016). Zinc finger protein ZBTB20 is an independent prognostic marker and promotes tumor growth of human hepatocellular carcinoma by repressing FoxO1. Oncotarget.

[67] Shen H., Luan F., Liu H., Gao L., Liang X., Zhang L., Sun W., Ma C. (2008). ZHX2 is a repressor of alpha-fetoprotein expression in human hepatoma cell lines. J. Cell Mol. Med..

[68] Marchio A., Bertani S., Rojas Rojas T., Doimi F., Terris B., Deharo E., Dejean A., Ruiz E., Pineau P. (2014). A peculiar mutation spectrum emerging from young peruvian patients with hepatocellular carcinoma. PLoS ONE.

[69] Shen S., Feng H., Liu L., Su W., Yu L., Wu J. (2020). TCP10L negatively regulates alpha-fetoprotein expression in hepatocellular carcinoma. BMB Rep..

[70] Ogden S.K., Lee K.C., Wernke-Dollries K., Stratton S.A., Aronow B., Barton M.C. (2001). p53 targets chromatin structure alteration to repress alpha-fetoprotein gene expression. J. Biol. Chem..

[71] Nguyen T.T., Cho K., Stratton S.A., Barton M.C. (2005). Transcription factor interactions and chromatin modifications associated with p53-mediated, developmental repression of the alpha-fetoprotein gene. Mol. Cell Biol..

[72] Peng S.Y., Chen W.J., Lai P.L., Jeng Y.M., Sheu J.C., Hsu H.C. (2004). High alpha-fetoprotein level correlates with high stage, early recurrence and poor prognosis of hepatocellular carcinoma: Significance of hepatitis virus infection, age, p53 and beta-catenin mutations. Int. J. Cancer.

[73] Ogden S.K., Lee K.C., Barton M.C. (2000). Hepatitis B viral transactivator HBx alleviates p53-mediated repression of alpha-fetoprotein gene expression. J. Biol. Chem..

[74] Ye G., Sun G., Cheng Z., Zhang L., Hu K., Xia X., Zhou Y. (2017). p55PIK regulates alpha-fetoprotein expression through the NF-κB signaling pathway. Life Sci..

[75] Li M.S., Li P.F., He S.P., Du G.G., Li G. (2002). The promoting molecular mechanism of alpha-fetoprotein on the growth of human hepatoma Bel7402 cell line. World J. Gastroenterol..

[76] Li M., Zhu M., Li W., Lu Y., Xie X., Wu Y., Zheng S. (2013). Alpha-fetoprotein receptor as an early indicator of HBx-driven hepatocarcinogenesis and its applications in tracing cancer cell metastasis. Cancer Lett..

[77] Esteban C., Terrier P., Frayssinet C., Uriel J. (1996). Expression of the alpha-fetoprotein gene in human breast cancer. Tumour. Biol..

[78] Ogunwobi O.O., Harricharran T., Huaman J., Galuza A., Odumuwagun O., Tan Y., Ma G.X., Nguyen M.T. (2019). Mechanisms of hepatocellular carcinoma progression. World J. Gastroenterol..

[79] Toder V., Blank M., Gold-Gefter L., Nebel L. (1983). The effect of alpha-fetoprotein on the growth of placental cells in vitro. Placenta.

[80] Semenkova L.N., Dudich E.I., Dudich I.V. (1997). Induction of apoptosis in human hepatoma cells by alpha-fetoprotein. Tumour. Biol..

[81] Li M.S., Li P.F., Chen Q., Du G.G., Li G. (2004). Alpha-fetoprotein stimulated the expression of some oncogenes in human hepatocellular carcinoma Bel 7402 cells. World J. Gastroenterol..

[82] Sporn M.B., Roberts A.B. (1988). Peptide growth factors are multifunctional. Nature.

[83] Dudich E., Semenkova L., Gorbatova E., Dudich I., Khromykh L., Tatulov E., Grechko G., Sukhikh G. (1998). Growth-regulative activity of human alpha-fetoprotein for different types of tumor and normal cells. Tumour. Biol..

[84] Keel B.A., Eddy K.B., Cho S., May J.V. (1991). Synergistic action of purified alpha-fetoprotein and growth factors on the proliferation of porcine granulosa cells in monolayer culture. Endocrinology.

[85] Li M., Li H., Li C., Wang S., Jiang W., Liu Z., Zhou S., Liu X., McNutt M.A., Li G. (2011). Alpha-fetoprotein: A new member of intracellular signal molecules in regulation of the PI3K/AKT signaling in human hepatoma cell lines. Int. J. Cancer.

[86] Zhu M., Lin B., Zhou P., Li M. (2015). Molecular Analysis of AFP and HSA Interactions with PTEN Protein. Biomed. Res. Int..

[87] Dudich E., Semenkova L., Dudich I., Gorbatova E., Tochtamisheva N., Tatulov E., Nikolaeva M., Sukhikh G. (1999). alpha-fetoprotein causes apoptosis in tumor cells via a pathway independent of CD95, TNFR1 and TNFR2 through activation of caspase-3-like proteases. Eur. J. Biochem..

[88] Semenkova L.N., Dudich E.I., Dudich I.V., Shingarova L.N., Korobko V.G. (1997). Alpha-fetoprotein as a TNF resistance factor for the human hepatocarcinoma cell line HepG2. Tumour. Biol..

[89] Semenkova L., Dudich E., Dudich I., Tokhtamisheva N., Tatulov E., Okruzhnov Y., Garcia-Foncillas J., Palop-Cubillo J.A., Korpela T. (2003). Alpha-fetoprotein positively regulates cytochrome c-mediated caspase activation and apoptosome complex formation. Eur. J. Biochem..

[90] Dudich E., Semenkova L., Dudich I., Denesyuk A., Tatulov E., Korpela T. (2006). Alpha-fetoprotein antagonizes X-linked inhibitor of apoptosis protein anticaspase activity and disrupts XIAP-caspase interaction. FEBS J..

[91] Yang X., Zhang Y., Zhang L., Zhang L., Mao J. (2008). Silencing alpha-fetoprotein expression induces growth arrest and apoptosis in human hepatocellular cancer cell. Cancer Lett..

[92] Li M., Li H., Li C., Zhou S., Guo L., Liu H., Jiang W., Liu X., Li P., McNutt M.A. (2009). Alpha fetoprotein is a novel protein-binding partner for caspase-3 and blocks the apoptotic signaling pathway in human hepatoma cells. Int. J. Cancer.

[93] Lin B., Zhu M., Wang W., Li W., Dong X., Chen Y., Lu Y., Guo J., Li M. (2017). Structural basis for alpha fetoprotein-mediated inhibition of caspase-3 activity in hepatocellular carcinoma cells. Int. J. Cancer.

[94] Li M., Zhou S., Liu X., Li P., McNutt M.A., Li G. (2007). alpha-Fetoprotein shields hepatocellular carcinoma cells from apoptosis induced by tumor necrosis factor-related apoptosis-inducing ligand. Cancer Lett..

[95] Crainie M., Semeluk A., Lee K.C., Wegmann T. (1989). Regulation of constitutive and lymphokine-induced Ia expression by murine alpha-fetoprotein. Cell. Immunol..

[96] Nicholas N.S., Panayi G.S. (1986). Immunosuppressive properties of pregnancy serum on the mixed lymphocyte reaction. Br. J. Obstet. Gynaecol..

[97] Um S.H., Mulhall C., Alisa A., Ives A.R., Karani J., Williams R., Bertoletti A., Behboudi S. (2004). Alpha-fetoprotein impairs APC function and induces their apoptosis. J. Immunol..

[98] Li M., Liu X., Zhou S., Li P., Li G. (2005). Effects of alpha fetoprotein on escape of Bel 7402 cells from attack of lymphocytes. BMC Cancer.

[99] Li Q.T., Qiu M.J., Yang S.L., Fang X., He X.X., Wang M.M., Li Y.N., Xiong Z.F., Huang S. (2020). Alpha-Fetoprotein Regulates the Expression of Immune-Related Proteins through the NF-κB (P65) Pathway in Hepatocellular Carcinoma Cells. J. Oncol..

[100] Zahran A.M., Nafady-Hego H., Mansor S.G., Abbas W.A., Abdel-Malek M.O., Mekky M.A., Hetta H.F. (2019). Increased frequency and FOXP3 expression of human CD8(+)CD25(High.+) T lymphocytes and its relation to CD4 regulatory T cells in patients with hepatocellular carcinoma. Hum. Immunol..

[101] Esteban C., Trojan J., Macho A., Mishal Z., Lafarge-Frayssinet C., Uriel J. (1993). Activation of an alpha-fetoprotein/receptor pathway in human normal and malignant peripheral blood mononuclear cells. Leukemia.

[102] Zhou S., Venkatramani R., Gupta S., Wang K., Stein J.E., Wang L., Mascarenhas L. (2017). Hepatocellular malignant neoplasm, NOS: A clinicopathological study of 11 cases from a single institution. Histopathology.

[103] Uchino K., Tateishi R., Shiina S., Kanda M., Masuzaki R., Kondo Y., Goto T., Omata M., Yoshida H., Koike K. (2011). Hepatocellular carcinoma with extrahepatic metastasis: Clinical features and prognostic factors. Cancer.

[104] Yoo D.J., Kim K.M., Jin Y.J., Shim J.H., Ko G.Y., Yoon H.K., Sung K.B., Lee J.L., Kang Y.K., Lim Y.S. (2011). Clinical outcome of 251 patients with extrahepatic metastasis at initial diagnosis of hepatocellular carcinoma: Does transarterial chemoembolization improve survival in these patients?. J. Gastroenterol. Hepatol..

[105] Katyal S., Oliver J.H., Peterson M.S., Ferris J.V., Carr B.S., Baron R.L. (2000). Extrahepatic metastases of hepatocellular carcinoma. Radiology.

[106] Yokoo T., Patel A.D., Lev-Cohain N., Singal A.G., Yopp A.C., Pedrosa I. (2017). Extrahepatic metastasis risk of hepatocellular carcinoma based on α-fetoprotein and tumor staging parameters at cross-sectional imaging. Cancer Manag. Res..

[107] Kumar S. (2018). Metastatic Recurrent Hepatocellular Carcinoma Post Liver Transplant. With Marked Pretransplant Elevation of Alpha Fetoprotein and No Evidence of Primary Neoplasm. Exp. Clin. Transplant..

[108] Hameed B., Mehta N., Sapisochin G., Roberts J.P., Yao F.Y. (2014). Alpha-fetoprotein level >1000 ng/mL as an exclusion criterion for liver transplantation in patients with hepatocellular carcinoma meeting the Milan criteria. Liver Transpl..

[109] Jin J., Niu X., Zou L., Li L., Li S., Han J., Zhang P., Song J., Xiao F. (2016). AFP mRNA level in enriched circulating tumor cells from hepatocellular carcinoma patient blood samples is a pivotal predictive marker for metastasis. Cancer Lett..

[110] Lu Y., Zhu M., Li W., Lin B., Dong X., Chen Y., Xie X., Guo J., Li M. (2016). Alpha fetoprotein plays a critical role in promoting metastasis of hepatocellular carcinoma cells. J. Cell. Mol. Med..

[111] Li Y., Tang Z.Y., Ye S.L., Liu Y.K., Chen J., Xue Q., Chen J., Gao D.M., Bao W.H. (2001). Establishment of cell clones with different metastatic potential from the metastatic hepatocellular carcinoma cell line MHCC97. World J. Gastroenterol..

[112] Koide N., Nishio A., Igarashi J., Kajikawa S., Adachi W., Amano J. (1999). Alpha-fetoprotein-producing gastric cancer: Histochemical analysis of cell proliferation, apoptosis, and angiogenesis. Am. J. Gastroenterol..

[113] Sheppard H.W., Sell S., Trefts P., Bahu R. (1977). Effects of alpha-fetoprotein on murine immune responses. I. Studies on mice. J. Immunol..

[114] Lutsenko S.V., Feldman N.B., Finakova G.V., Gukasova N.V., Petukhov S.P., Posypanova G.A., Skryabin K.G., Severin S.E. (2000). Antitumor activity of alpha fetoprotein and epidermal growth factor conjugates in vitro and in vivo. Tumour. Biol..

[115] Sotnichenko A.I., Severin S.E., Posypanova G.A., Feldman N.B., Grigor’ev M.I., Severin E.S., Petrov R.V. (1999). Water-soluble 2,3,7,8-tetrachlorodibenzo-p-dioxin complex with human alpha-fetoprotein: Properties, toxicity in vivo and antitumor activity in vitro. FEBS Lett..

[116] Moskaleva E.Y., Posypanova G.A., Shmyrev I.I., Rodina A.V., Muizhnek E.L., Severin E.S., Katukov V.Y., Luzhkov Y.M., Severin S.E. (1997). Alpha-fetoprotein-mediated targeting-a new strategy to overcome multidrug resistance of tumour cells in vitro. Cell Biol. Int..

[117] Ido A., Nakata K., Kato Y., Nakao K., Murata K., Fujita M., Ishii N., Tamaoki T., Shiku H., Nagataki S. (1995). Gene therapy for hepatoma cells using a retrovirus vector carrying herpes simplex virus thymidine kinase gene under the control of human alpha-fetoprotein gene promoter. Cancer Res..

[118] Zhu W., Peng Y., Wang L., Hong Y., Jiang X., Li Q., Liu H., Huang L., Wu J., Celis E. (2018). Identification of α-fetoprotein-specific T-cell receptors for hepatocellular carcinoma immunotherapy. Hepatology.

[119] Cai L., Caraballo Galva L.D., Peng Y., Luo X., Zhu W., Yao Y., Ji Y., He Y. (2020). Preclinical Studies of the Off.-Target. Reactivity of AFP(158)-Specific TCR Engineered T Cells. Front. Immunol..

[120] Liu H., Xu Y., Xiang J., Long L., Green S., Yang Z., Zimdahl B., Lu J., Cheng N., Horan L.H. (2017). Targeting Alpha-Fetoprotein (AFP)-MHC Complex. with CAR T-Cell Therapy for Liver Cancer. Clin. Cancer Res..

